# Low cost, p-ZnO/n-Si, rectifying, nano heterojunction diode: Fabrication and electrical characterization

**DOI:** 10.3762/bjnano.5.230

**Published:** 2014-11-24

**Authors:** Vinay Kabra, Lubna Aamir, M M Malik

**Affiliations:** 1 Nanotechnology Research Laboratory, Centre of Nanoscience and Engineering, Maulana Azad National Institute of Technology, Bhopal 462051, India

**Keywords:** capacitance–voltage measurements, current–voltage measurement, solution-processed rectifying p-ZnO/n-Si heterojunction diode, UV illumination

## Abstract

A low cost, highly rectifying, nano heterojunction (p-ZnO/n-Si) diode was fabricated using solution-processed, p-type, ZnO nanoparticles and an n-type Si substrate. p-type ZnO nanoparticles were synthesized using a chemical synthesis route and characterized by XRD and a Hall effect measurement system. The device was fabricated by forming thin film of synthesized p-ZnO nanoparticles on an n-Si substrate using a dip coating technique. The device was then characterized by current–voltage (*I*–*V*) and capacitance–voltage (*C*–*V*) measurements. The effect of UV illumination on the *I*–*V* characteristics was also explored and indicated the formation of a highly rectifying, nano heterojunction with a rectification ratio of 101 at 3 V, which increased nearly 2.5 times (232 at 3 V) under UV illumination. However, the cut-in voltage decreases from 1.5 V to 0.9 V under UV illumination. The fabricated device could be used in switches, rectifiers, clipper and clamper circuits, BJTs, MOSFETs and other electronic circuitry.

## Introduction

The fabrication of homo- and hetero-junction diodes based on nanomaterials is an emerging field that could allow for practical application of nanotechnology in electronics. The cost and performance of such devices are the most challenging tasks for the research community. Various techniques have been extensively employed to fabricate high performance, homo- and hetero-junctions based on various semiconductors. Among them, ZnO (with a high band gap of 3.37 eV) [1–2] has been recognized as one of the most popular semiconducting materials for device fabrication due to its excellent electrical and optical properties [3–4]. Much work has been demonstrated for heterojunctions based on n- and p-type ZnO nanoparticles using physical techniques [1–8] but the results were not satisfactory overall with respect to the rectification ratio and cut-in voltage [6–8]. Such physical techniques can certainly result in high performance diodes, however, the fabrication costs are very high, limiting their industrial applications. Therefore, there is a need for alternative, cost-effective methods to fabricate homo- and hetero-junction diodes based on semiconductor nanoparticles.

This research reports a strategy for fabrication of low cost, highly rectifying (p-ZnO/n-Si) nano heterojunction diode using solution-processed p-ZnO nanoparticles. The current–voltage (*I*–*V*) and capacitance–voltage (*C*–*V*) characteristics of heterojunctions were analyzed, resulting in rectification ratios of 101 and 232 (at 3 V) and cut-in voltages of 1.5 V and 0.9 V under dark and UV illumination, respectively. Additionally, the built-in potential was found to be 1.6 V. These results suggest that the device could be used in high voltage applications, which is an advantage compared to Si-based devices. UV illumination-dependent performance of the diode could also be utilized in space applications where wide band gap, semiconductor-based devices could perform better and may tolerate the extreme environment. The high rectification of the fabricated diode makes it applicable in all electronic circuitry, for example, switches, rectifiers, clipper and clamper circuits, BJTs and MOSFETs.

## Results and Discussion

### X-ray diffraction

**Figure 1:**
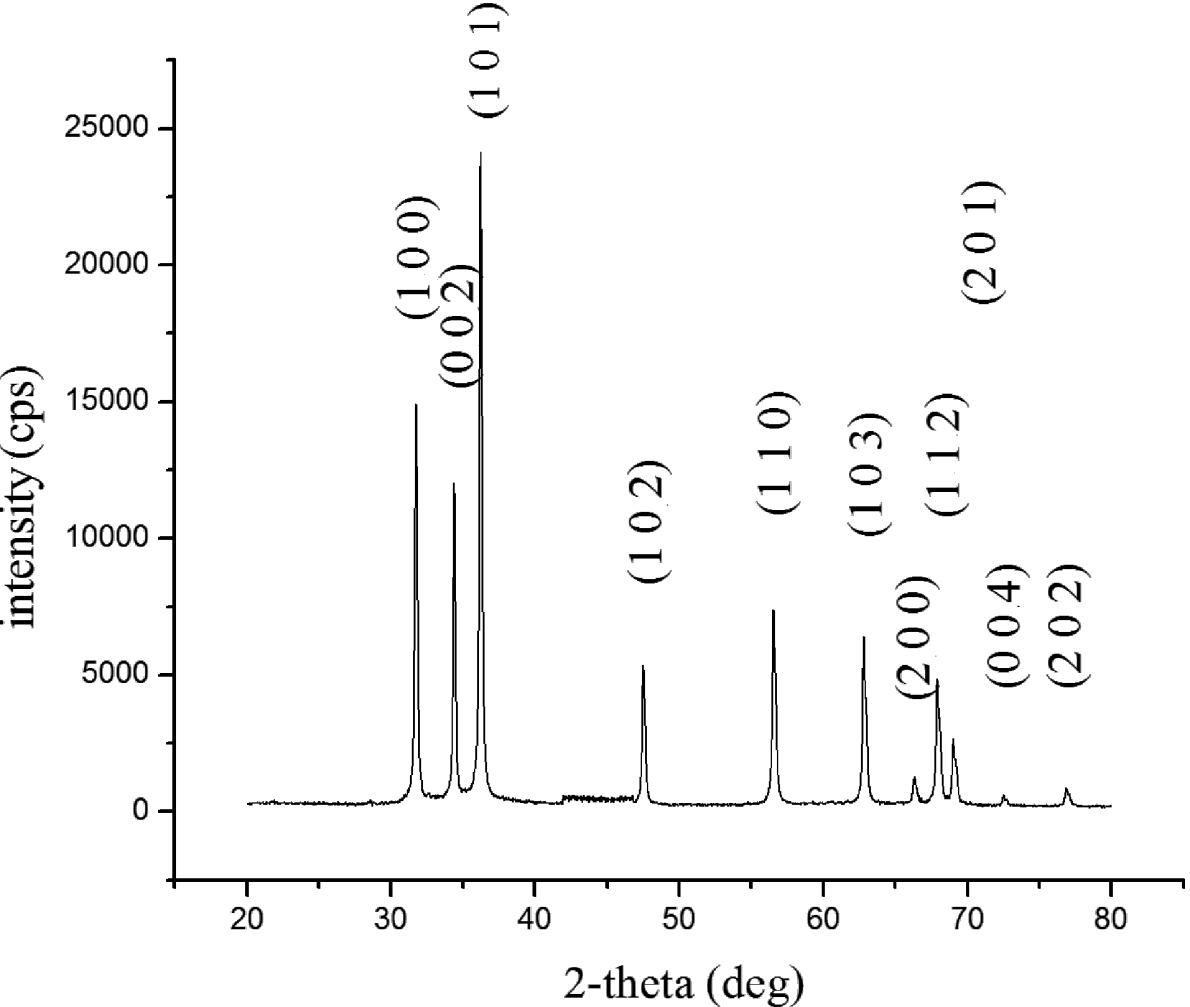
XRD pattern of p-ZnO nanoparticles.

Figure 1 shows the X-ray diffraction pattern of p-ZnO nanoparticles. The diffraction peaks of the sample correspond to the (100), (002), (101), (110), (103), and (112) planes of reflection for the hexagonal wurtzite structure of ZnO. All of the peaks are in good agreement with the JCPDS database file number 790208. The number of peaks observed in the XRD pattern indicates a polycrystalline nature of the ZnO [3]. The crystallite size was determined to be 26.07 nm using the Scherrer equation. The width of the diffraction peaks and crystallite size together indicate the formation of ZnO nanoparticles.

### Hall effect measurement

The Hall effect measurement of a p-ZnO rectangular pellet with dimensions 0.8 × 0.8 × 0.1 cm^3^ was performed using a four-probe van der Pauw method using silver contacts, and data were averaged to ensure accuracy. The carrier concentration, Hall mobility and resistivity of p-ZnO nanoparticles were found to be +5 × 10^14^ cm^−3^, 31.63 cm^2^/Vs, and 395.19 Ωcm, respectively. These results clearly indicate that the synthesized ZnO nanoparticles have p-type conductivity. A Hall measurement of the n-Si substrate was also performed on a silicon wafer with dimensions 1.4 × 0.9 × 0.04 cm^3^. The values of the carrier concentration, Hall mobility and resistivity of Si substrate were found to be 2.3 × 10^15^ cm^−3^, 555 cm^2^/Vs, and 5 Ωcm, respectively. It is apparent that the carrier concentration, mobility and resistivity of these p-ZnO nanoparticles are sufficient for use in the fabrication of a heterojunction diode. Furthermore, work is in progress to achieve a carrier concentration for the p-ZnO nanoparticles on the order of 10^18^ cm^−3^.

#### Current–voltage (*I*–*V*) characteristics

**Figure 2:**
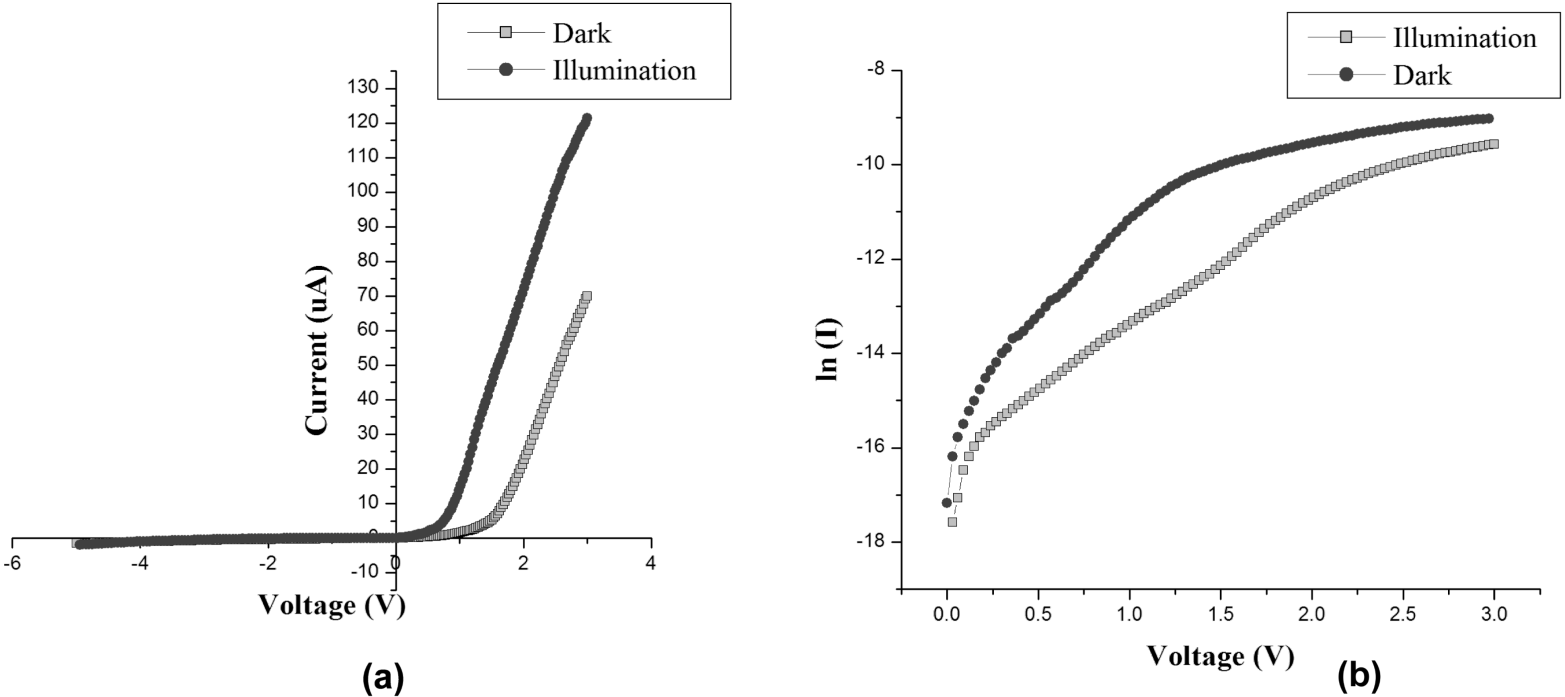
(a) *I–V* characteristics of the diode under dark and UV illumination and (b) ln*I* vs *V* curve under dark and UV illumination.

Figure 2a shows the *I*–*V* characteristics of the p-ZnO/n-Si nano heterojunction diode (area: 0.25 cm^2^) under dark and UV illumination (λ = 220 nm, intensity: 233 lux). It is clear from the *I*–*V* characteristics that the nano heterojunction possesses good rectification with a forward to reverse current ratio (*I*_F_/*I*_R_) of 101 under dark conditions, which increases to 232 under UV illumination at 3 V. These characteristics indicate a successful fabrication of a highly rectifying, nano heterojunction diode. The cut-in voltage was found to be 1.5 V under dark conditions, which decreases to 0.9 V under UV illumination. This information was extracted by extrapolating the linear portion of the graph to the *x*-axis. This change in the rectification ratio and cut-in voltage under dark and UV illumination is caused by the absorption of UV radiation by ZnO which produces extra electron–hole pairs. These extra electron–hole pairs then takes part in the current conduction process and increases the current exponentially in the forward bias [8]. On the other hand, in the reverse bias condition, the depletion width increases to produce a barrier in the flow of these photo-generated carriers. This effect, in turn, reduces the current and thus causes better rectification [8]. An increase in the current density from 0.28 mA/cm^2^ (dark) to 0.5 mA/cm^2^ (UV illumination) was observed. The reverse breakdown voltage of the fabricated device is very high (greater than 100 V). This was not evidenced here due to limitations in instrumentation. The reason for such a high breakdown voltage is attributed to the carrier concentration (10^14^ to 10^15^ cm^−3^) of the p-ZnO nanoparticles [9]. The current–voltage relation for a real diode is expressed as [1,9–10]:

[1]
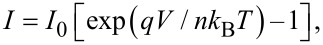


where, *I*_0_ is reverse saturation current, *V* is the forward voltage, *k*_B_ is the Boltzmann constant, *q* is the electric charge carried by a single electron, *T* is the temperature and *n* is the ideality factor. The values of *I*_0_ and *n* were determined from the ln *I*–*V* plot (Figure 2b). The slope of the curve gives the ideality factor (*n*) [1,9–10] and intercept at the *y*-axis (after extrapolating the linear portion of the curve) gives the value of the reverse saturation current *I*_0_ [9–10]. The values for *I*_0_ and *n* were found to be 5.36 × 10^−8^ A and 2.78, respectively (between 0 to 1.5 V) for dark conditions [1,9–10] and 8.42 × 10^−8^ A and 2.98, respectively (between 0 to 1 V) under UV illumination. At higher voltages (2–3 V), the value of *n* was found to be ≈1. These results clearly depict that the recombination current dominates over the diffusion current at lower voltages, while the diffusion current dominates over the recombination current at higher voltages (2–3 V), as expected from an ideal diode. In this case, the recombination is dominated by Auger recombination, as expected from any highly doped semiconductor (due to the Si substrate) junction [9]. Therefore, it was concluded that the p-ZnO/n-Si nano heterojunction behaves as a normal diode with a high breakdown voltage, good rectification, and UV-enhanced performance. These features can be utilized in space applications where silicon or GaAs-based devices cannot be implemented.

#### Capacitance–voltage characteristics

**Figure 3:**
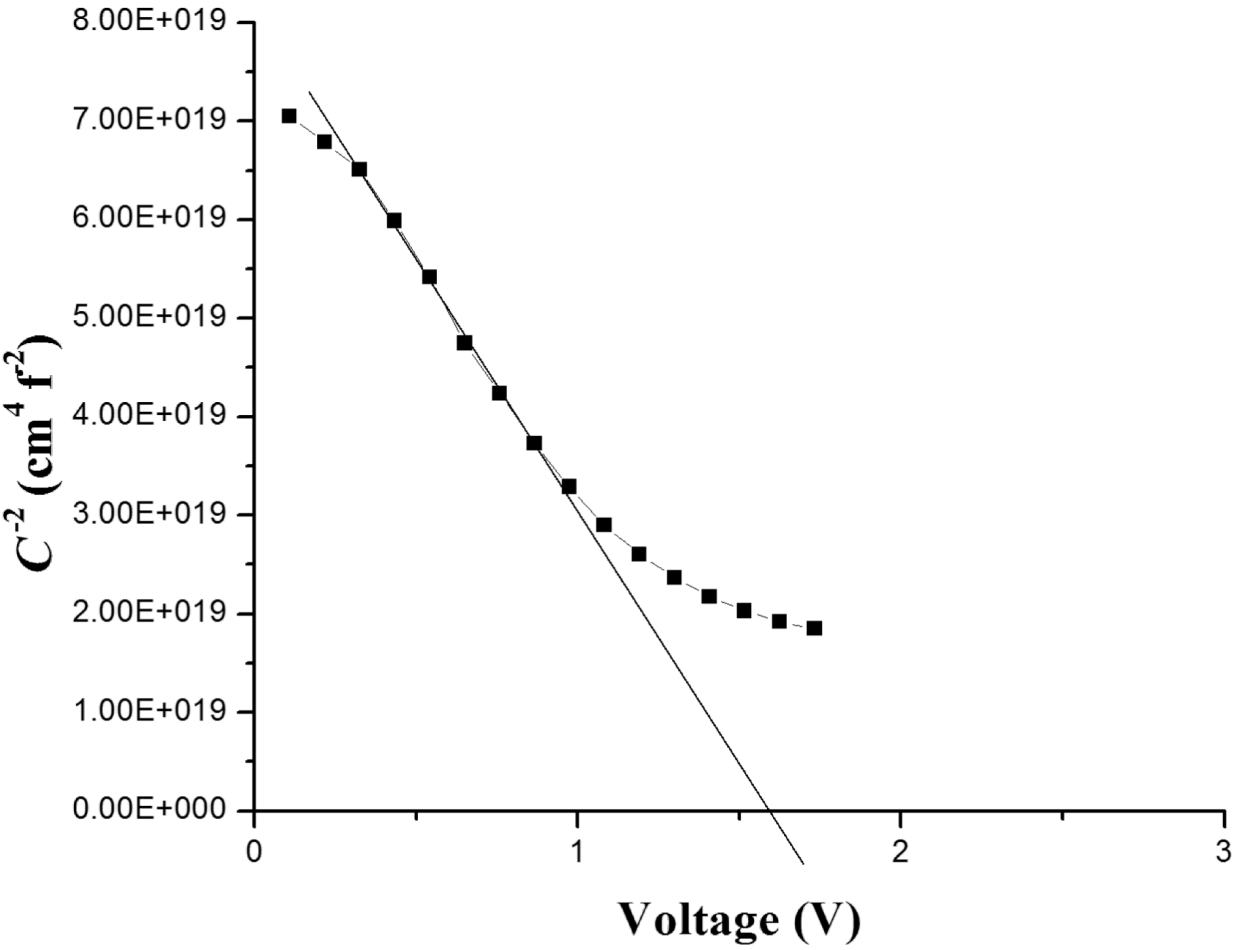
1/*C*^2^ versus voltage curve of the nano heterojunction diode.

Figure 3 shows the 1/*C*^2^–voltage characteristics of the nano heterojunction observed at 100 kHz AC frequency with an amplitude of 1 V. It can be seen from the figure that as the forward bias voltage increases, 1/*C*^2^ decreases and reaches its minimum value at the built-in voltage. The extension of the 1/*C*^2^–voltage curve to 1/*C*^2^ = 0 gives the built-in voltage [3,9–10], which was found to be 1.6 V (Figure 3). This high value for the built-in voltage is assigned to the low intrinsic carrier concentration of p-ZnO. Since the band gap of p-ZnO is high (3.37 eV) (which is related to the band gap of the material as given in Equation 2), the intrinsic carrier concentration will be low for ZnO. This high built-in voltage is the origin of the high cut-in voltage of the fabricated nano heterojunction diode and can be calculated as:

[3]
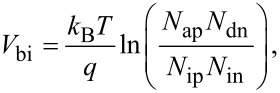


where *N*_ap_ and *N*_dn_, and *N*_ip_ and *N*_in_ are the carrier concentrations and intrinsic carrier concentrations of p-ZnO and n-Si, respectively, and

[2]
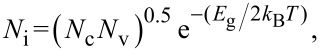


where *N*_c_ and *N*_v_ are the material constants.

The total depletion width, the depletion width for the n- and p-side, and the maximum electric field at zero bias are calculated using Equations 4–6 [9–10] as follows:

[4]
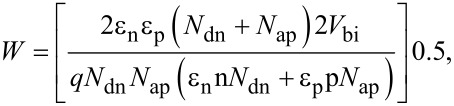


[5]
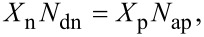


[6]
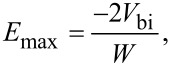


where *V*_bi_ is the built-in voltage, *X*_n_ and *X*_p_ are the depletion width for the n- and p-side, and ε_n_ and ε_p_ are the dielectric constants of n-Si and p-ZnO, respectively. The dielectric constants ε_p_ and ε_n_ were found to be 7 and 11.7, respectively, as derived from impedance spectroscopy [9–10]. The depletion width on the n-side is found to be shorter than on the p-side because the carrier concentration of n-Si is higher than p-ZnO, which is supported by the Hall effect results. The calculated values of these various diode parameters using Equations 4–6 are listed in Table 1.

**Table 1:** Values of several diode parameters calculated from the *C*–*V* analysis.

Diode parameter	Values

*W* = *X*_n_ + *X*_p_	1.8 µm
*X*_n_	0.32 µm
*X*_p_	1.48 µm
*E*_max_	1.78 × 10^4^ V/cm

#### Energy band diagram and carrier transport

**Figure 4:**
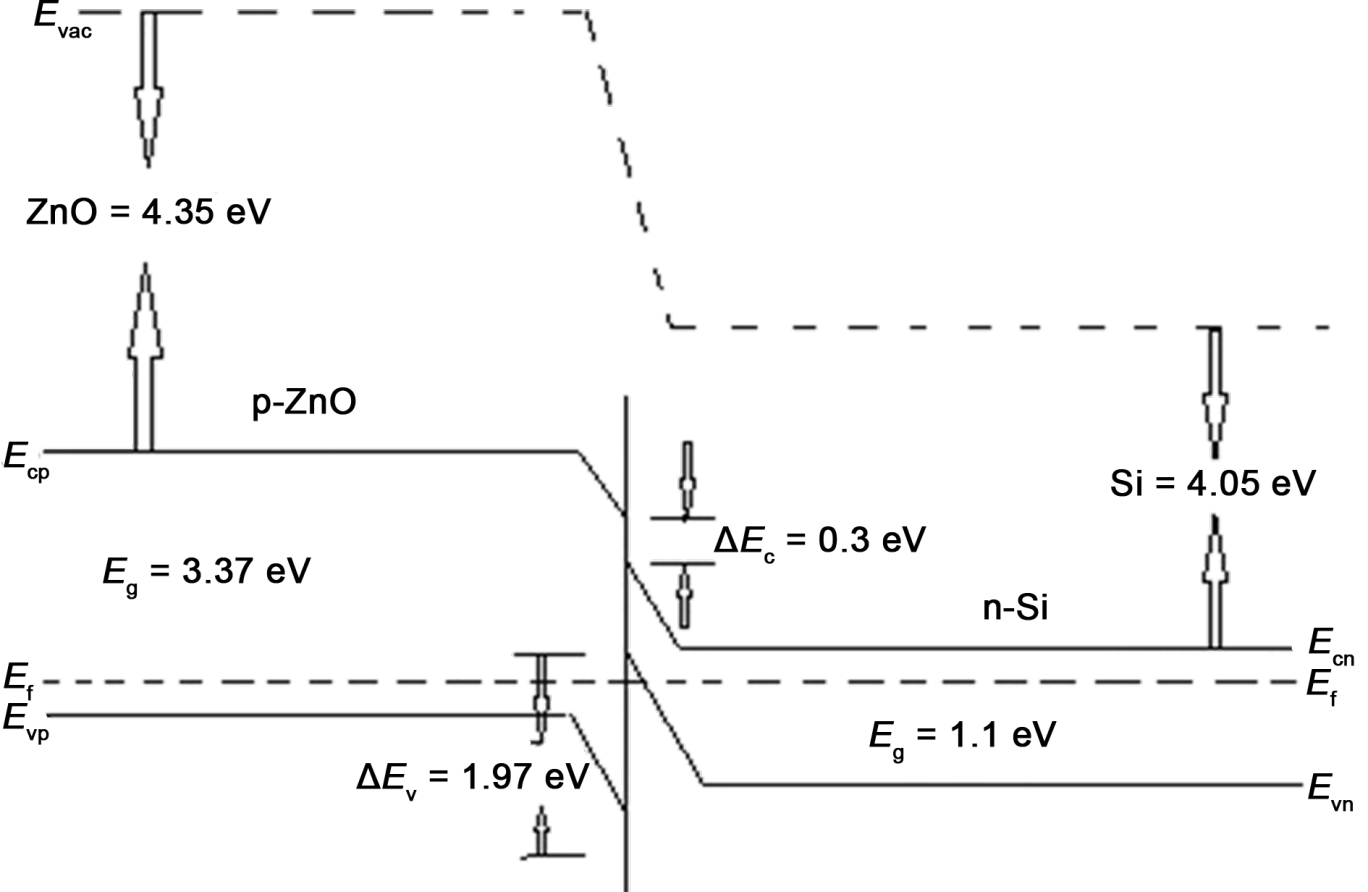
Band diagram of a p-ZnO/n-Si nano heterojunction diode.

The energy band diagram of the p-ZnO/n-Si nano heterojunction diode is depicted in Figure 4. The band gap of n-Si is 1.1 eV [9–10] and p-ZnO is 3.37 eV and the electron affinity of p-ZnO (χ_p_) and n-Si (χ_n_) is 4.35 eV and 4.05 eV, respectively [8]. The energy band diagram shows a small conduction band offset of 0.3 eV as calculated by Δ*E*_c_ = *q*(χ_p −_ χ_n_) and a large valance band offset 1.97 eV calculated by Δ*E*_v_ = Δ*E*_g_ − Δ*E*_c_. There is a diffusion of electrons from n-Si to p-ZnO and a diffusion of holes from p-ZnO to n-Si. At low, forward voltage, the current is limited by a space charge region, however, by increasing the forward voltage, the depletion width decreases and current increases exponentially, following Equation 1.

## Conclusion

A low cost, highly rectifying, p-ZnO/n-Si nano heterojunction diode was fabricated using solution-processed, p-ZnO nanoparticles and a n-Si substrate. The *I*–*V* characteristics of nano heterojunction were analyzed under dark and UV illumination, and an increase in the rectification ratio and a decrease in the cut-in voltage under UV illumination were observed. The high rectification, high cut-in voltage, and UV-enhanced performance of the fabricated diode renders it highly relevant for space applications and voltage regulators, where wide band gap, semiconductor-based devices might perform better and tolerate the extreme environment. These results are promising and offer the prospect of fabrication of low cost diodes using solution-processed nanoparticles for high voltage applications. This is in obvious contrast to Si-based devices, which cannot endure such conditions. Such a high rectification presented by the nano heterojunction diode will generally be useful in all electronic circuitry, for example, switches, rectifiers, clipper and clamper circuits, etc. However, there is still progress to be made on this nano heterojunction for further application.

## Experimental

### Synthesis of p-type ZnO nanoparticles

For the synthesis of p-type ZnO (p-ZnO) nanoparticles by a chemical route, 200 mL of aqueous zinc acetate solution (25 mM) was mixed with a 25% aqueous ammonia solution and aluminum chloride as nitrogen and aluminum sources, respectively. These were mixed in the atomic ratio of Zn:N:Al to 1:0.06:0.03 at room temperature under constant stirring. A freshly prepared tetramethylammonium hydroxide (TMAH) solution was added to the above mixture. The mixture was then left at 70 °C for 30 min under constant stirring. After some time, the color of the mixture turned milky white. White precipitates were then extracted after washing several times with distilled water. Parallel experiments were also conducted for different concentrations of dopant, but these results were not suitable for the above atomic ratio, which was determined after optimization.

#### Device fabrication

**Figure 5:**
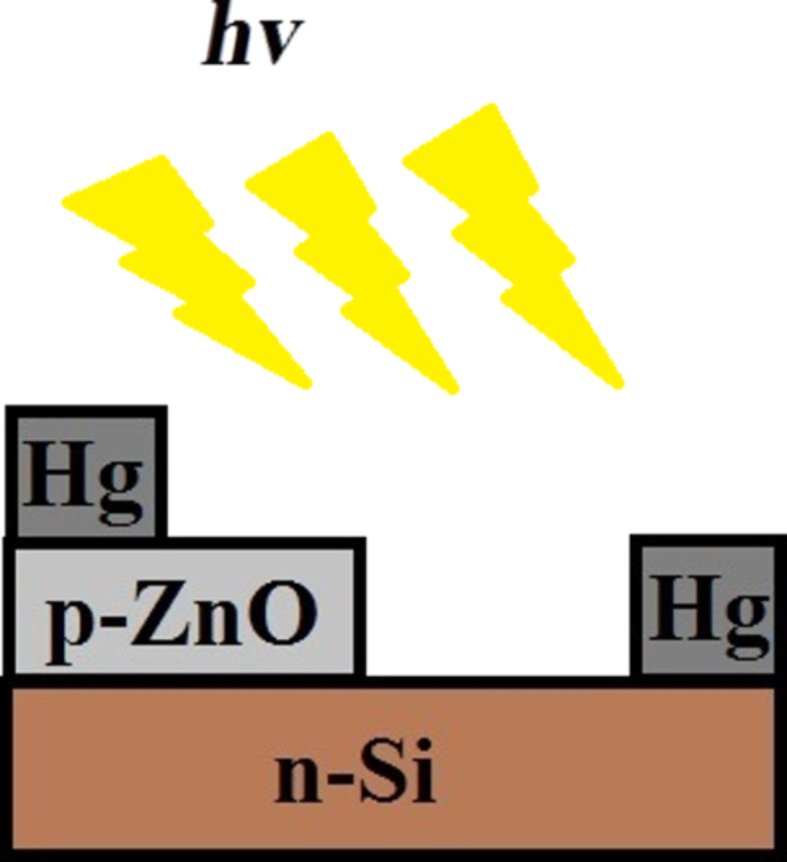
Layout of device.

The p-type ZnO thin film was formed on the n-type Si substrate using a dip coating technique with an immersion rate of 9 mm/s, a dwell time of 20 s, and a withdrawal rate of 1 mm/s, with consecutive drying for 99 s at 50 °C. This process was repeated several times to obtain a film thickness of 14 µm. The film was then annealed at 500 °C for 2 h. Mercury contacts were then formed over the n-Si substrate and the p-ZnO film as indicated in Figure 5. Mercury was used to eradicate any possibility of rectification through the contacts, as its work function (4.5 eV) is higher than that of p-ZnO (<4 eV). The mercury contacts are assumed to form ohmic contacts [9].

#### Characterization

A Rigaku Minflex-2 X-ray diffractometer was used for determination of the crystalline phase of the p-ZnO nanoparticles. The Hall effect measurement system (ECOPIA, model HMS-3000) was used for electrical characterization of the sample. An electrometer (KEITHLEY, 6517B) was used for the current–voltage (*I*–*V*) measurements of the diode and an impedance analyzer (WAYNE KERR, 6500B) was used for the capacitance–voltage (*C*–*V*) measurements of the iode.
